# Neuroimaging findings in DYT1 dystonia and the pathophysiological implication: A systematic review

**DOI:** 10.1002/brb3.3023

**Published:** 2023-05-11

**Authors:** Funmilola T. Taiwo, Philip B. Adebayo

**Affiliations:** ^1^ Neurology Unit, Department of Medicine University College Hospital Ibadan Nigeria; ^2^ Neurology Section, Department of Internal Medicine Aga Khan University Dar es Salaam Tanzania

**Keywords:** dystonia, DYT1, hypothesis, neuroimaging, pathophysiology

## Abstract

**Background:**

Primary generalized dystonia due to the DYT1 gene is an autosomal dominant disorder caused by a GAG deletion on chromosome 9q34. It is a well‐defined, genetically proven, isolated dystonia syndrome. However, its pathophysiology remains unclear.

**Objectives:**

This study was aimed at profiling the functional neuroimaging findings in DYT1 dystonia and harmonizing the pathophysiological implications for DYT1 dystonia from the standpoint of different neuroimaging techniques.

**Methods:**

A systematic review was conducted using identified studies published in English from Medline, PsycINFO, Embase, CINAHL, and the Cochrane Database of Systematic Reviews (CDSR), between 1985 and December 2019 (PROSPERO protocol CRD42018111211).

**Results:**

All DYT1 gene carriers irrespective of clinical penetrance have reduced striatal GABA, dopamine receptors and increased metabolic activity in the lentiform nucleus, supplementary motor area, and cerebellum in addition to an abnormal cerebellothalamocortical pathway. Nonmanifesting carriers on the other hand have a disruption of the distal (thalamocortical) segment and have larger putaminal volumes than manifesting carriers and healthy controls. Activation of the midbrain, thalamus, and sensorimotor cortex was only found in the manifesting carriers.

**Conclusions:**

Therefore, we propose that DYT1 dystonia is a cerebellostriatothalamocortical network disorder affecting either the structure or function of the different structures or nodes in the network.

## Graphical abstract

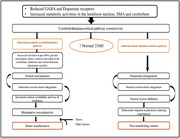

## 1 INTRODUCTION

Hereditary dystonia due to the DYT1 gene mutation is the most studied and well‐characterized form of primary generalized dystonia. It is caused by a deletion of the GAG trinucleotide in the DYT1 gene on chromosome 9q32–q34 encoding torsinA (Ozelius et al., 1989). It has an age‐dependent phenotypic penetrance of 25%−30% and a marked variability in clinical presentation and disease severity (Bressman et al., 1994; Opal et al., 2002). Although DYT1 dystonia is the most studied of the genetically proven primary generalized dystonia, its pathophysiology remains unclear. Some researchers proposed a neurodevelopmental and neurodegenerative mechanism (Carbon et al., 2010; McNaught et al., 2004; Niethammer et al., 2011), because most patients with the DYT1 dystonia have their onset in childhood or early adolescence, when the maturation of thalamic and sensorimotor cortical pathways is known to occur (Barnea‐Goraly et al., 2005; Gogtay et al., 2004; Paus, 2005).

The definition of primary dystonia implies a normal brain structure (Eidelberg et al., 1998). Albeit recent advances in radiological techniques have consistently demonstrated abnormalities in the structure and function of the dystonic brain. Neuroimaging has been used to study the primary focal dystonias and different aspects of their pathophysiology; however, most previous pathophysiological works in primary generalized dystonia have focused on neurophysiology. The first link to the basal ganglia as the anatomical basis of dystonia was described over a century ago (Neychev et al., 2008). However, there is a paradigm shift now, where dystonia is being viewed as a motor circuit disorder, wherein there is a network with multiple nodes; each node represents different structures in the brain. Dystonia can thus result from dysfunction of specific nodes in the network or an aberrant communication between the nodes. This study aims to identify the different nodes in this network model and how they contribute to disease manifestation or carrier state in DYT1 dystonia.

## 2 METHODS

We conducted this review in accordance with the Preferred Reporting Items for systematic reviews and meta‐analysis (PRISMA‐P). A pilot search to identify appropriate search terms was first conducted.

### 2.1 Data sources and searches

**FIGURE 1:**
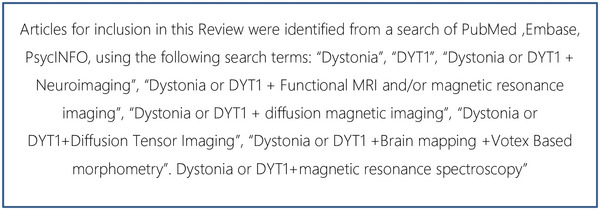
Search terms.

An exhaustive electronic and hand search for all relevant studies (published and unpublished) was then performed for studies from 1985 to December 2019. Search was restricted to studies published in English language. The following major databases were searched: Medline, Embase, PsycINFO, CINAHL, and the Cochrane Database of Systematic Reviews (CDSR). Content experts who were principal investigators of relevant studies were contacted via email when necessary. Search terms used include DYT1, dystonia, AND, neuroimaging, Voxel‐based morphometry (VBM), Positron emission tomography (PET), Single‐photon emission computed tomography (SPECT), Diffusion Tensor Imaging (DTI), Magnetic resonance imaging (MRI), functional Magnetic resonance imaging (fMRI), tomography, and Magnetic Resonance Spectroscopy (MRS) (Figure 1). Snowballing was performed for review articles. Finally, an RSS feed was set up for any article on imaging in dystonia to avoid missing those published after the search.

### 2.2 Study selection

**FIGURE 2:**
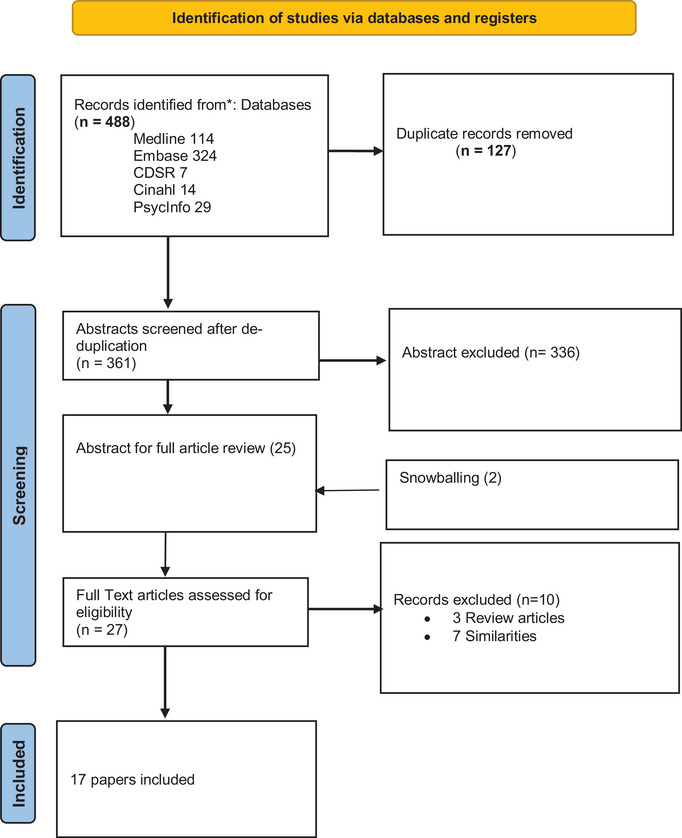
Prisma flow chart.

Studies were included if the participants had the DYT1 gene, whether they manifested the disease or not, and comparator were healthy individuals or nongenetic forms of primary dystonia or other genetic forms of primary dystonia. Titles and abstracts were independently screened against eligibility criteria by both authors (FTT, PBA). Disagreements were resolved by discussion. Full texts of screened papers were assessed for inclusion criteria. The flow diagram of study selection and reasons for exclusion to conform to the PRIMSA statement is presented in Figure 2.

### 2.3 Data extraction, synthesis, and analysis

A data extraction algorithm sheet was developed. Selected articles were sorted first according to authors’ name, then according to imaging modality used (to avoid double‐counting). We realized that same set of subjects were used for different neuroimaging modalities while the authors reported different aspect of the neuroimaging studies. We therefore chose a single most representative article, which reports all the findings of a chosen imaging modality, for example, fluorodeoxyglucose positron emission tomography (FDG/PET). The outcomes of consideration in the selected studies were the types of neuroimaging abnormalities demonstrated among manifesting and nonmanifesting carriers of DYT1 gene. The heterogeneity of the studies and outcomes precludes a meta‐analysis. Therefore, for the purpose of this review, a narrative synthesis of the findings, using a textual approach is as presented.

## 3 RESULTS

**FIGURE 3:**
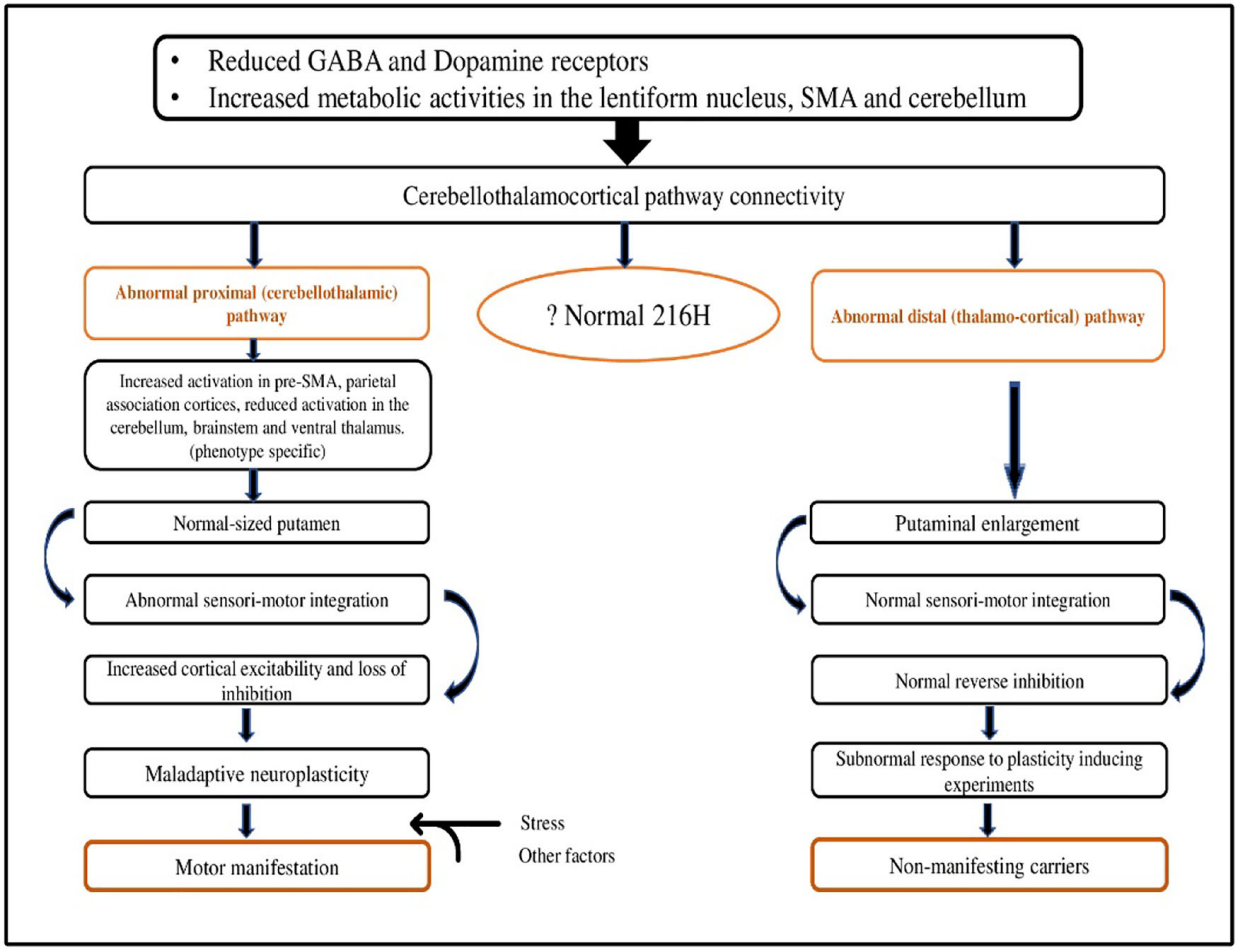
Genetic Susceptibility in DYT1. A hypothesis on the pathophysiology of DYT1 primary generalized dystonia. Genetic susceptibility evidenced by a carrier state of the DYT1 GAG deletion is associated with specific metabolic changes in the certain areas of the brain, and a reduction in the availability of gamma aminobutyric acid (GABA) and D2 receptors in the striatum. This is associated with an abnormality of the cerebellothalamocortical pathway. This pathway is divided into a proximal (cerebello‐thalamic), and a distal (thalamocortical). Abnormality affecting the proximal part alone, was demonstrated in manifesting carriers and is associated with a concomitant increase in activation of the supplementary motor area (SMA). Abnormality affecting both the proximal and distal was found in nonmanifesting carriers and this is said to prevent the transmission of abnormal impulses to the cortex and hence the motor consequence of the dystonia. A normal fractional anisotropy, in the region of the superior cerebellar peduncle was found in a patient with the 216H polymorphism and suggested to be associated with a normal cerebellothalamocortical pathway. The increase activation of the sensorimotor cortex which was found to be of a higher magnitude in Manifesting DYT1(MDYT1) is associated with abnormal sensorimotor integration and leads to cortical hyperexcitability and loss of inhibition and hence maladaptive neuroplasticity and consequently dystonia. The Nonmanifesting DYT1 (NMDYT1), on the other hand, in addition to the abnormality of the thalamocortical segment, have enlargement of the putamen, which appears to be compensatory. Sensorimotor integration, in them is said to compare with that of normal controls and hence, they do not have cortical hyperexcitability of a magnitude that is enough to cause maladaptive neuroplasticity. Additionally, the larger putaminal volume appears to prevent the development of maladaptive neuroplasticity, as this cohort of subjects were noted to have even subnormal response to plasticity‐inducing experimental protocols like repetitive transcranial magnetic stimulation (rTMS) and inhibitory paired associative stimulation (IPAS) and hence do not develop dystonia.

A total of 17 articles were included in this study, as highlighted in the Prisma flowchart (Figure 2). All studies selected are cross‐sectional, case‐control studies published in English. The studies included 34 manifesting carriers of the DYT1 (MDYT1) mutation, 33 nonmanifesting carriers of the DYT1 (NMDYT1) mutation, 10 nonmanifesting carriers of the DYT6 (NMDYT6) mutation, 15 manifesting carriers of DYT6 (MDYT6) mutation, 34 participants with the sporadic form of primary dystonia, and 100 healthy control subjects. The main inclusion criteria are as shown in the Prisma flowchart. Studies included male and female participants with ages ranging from 18 to 72 years and disease duration ranging from 15 to 54 years. Medication use comprises trihexyphenidyl, tetrabenazine, botulinum toxin, clonazepam, and baclofen. All the studies employed the above‐mentioned neuroimaging modalities, and the outcomes being considered are morphological and structural changes, alteration in glucose metabolism or regional cerebral blood flow, and receptor availability. No unpublished relevant studies were obtained. The heterogeneity of the studies and the outcomes thereof precluded a meta‐analysis; hence, a narrative synthesis of the extant literature in a textual approach is presented. Table 1 summarizes the studies and the findings. Figure 3 encapsulates our proposed model of genetic susceptibility in DYT1.

**TABLE 1:** Summary of neuroimaging findings in DYT1 dystonia.

First author, year	Country	Population/setting	Sample size	Controls	Age range (years)	Aim	Mutation (DYT1) carriers no:	Imaging technique	Reported outcome
Whone 2004	UK	Case‐control/outpatient	22	15	33−66	To investigate if an alteration in basal ganglia opioid binding was present in DYT1 primary torsion dystonia (PTD)	7MDYT1	[^11^C]‐diprenorphine/**PET**	No difference in regional mean [^11^C]‐diprenorphine binding was found between DYT1‐PTD and controls, and no correlation between the severity of dystonia and opioid binding was seen
Carbon 2009	US	Case‐control/outpatient	46	13	16−55	To determine whether changes in D2 receptor availability are present in carriers of genetic mutations for primary dystonia	12NMDYT1 9MDYT1 4NMDYT6 8MDYT6	[^11^C]‐raclopride/**PET**	Significant reductions in striatal and thalamic D2 receptor availability were evident in both groups of mutation carriers relative to healthy controls (*p* < .001)
Garibotto 2011	Italy	Case‐control/outpatient	25	11	28−56	In vivo assessment of the GABAergic system in dystonia	9MDYT1 5SPORADIC	[^11^C]‐flumazenil/**PET**	A reduction in GABAa receptor expression/affinity both in DYT1 carriers and sporadic patients in primary motor and premotor cortex, in primary and secondary somatosensory cortex, and in the motor component of the cingulate gyrus
Eidelberg 1998	USA	Case‐control	31	14	16−67	To determine the metabolic substrates of brain dysfunction in DYTl dystonia	7NMDYT1 10MDYT1	[^18^F]‐FDG/**PET**	Hypermetabolism in the lentiform nuclei, Cb, and SMA in all mutation carriers and persisted in MDYT1 in sleep when movements were suppressed Hypermetabolism in the midbrain, cerebellum, and thalamus in MDYT1 and significantly suppressed in sleep (MR pattern)
Carbon 2004	USA	Case‐control	47	11	16−67	To identify specific regions that discriminated subjects according to clinical penetrance and genotype	12NMDYT1 11MDYT1 5NMDYT6 7MDYT6	[^18^F]‐FDG/**PET**	Hypermetabolism in the putamen anterior cingulated and inferior cerebellum in all mutation carriers Bilateral hypermetabolism in the pre‐supplementary motor area parietal association cortices in MDYT1 compared to NMDYT1
Ghilardi 2003	USA	Case‐control	24	12	16−67	To determine whether motor behavior is impaired in these subjects	12NMDYT1	H2^[15]^O/**PET**	Increased rCBF in the left premotor cortex and right SMA and a concomitant reduction in the Cb during motor execution (no significant difference between mutation carriers and controls) Increased rCBF in the left ventral prefrontal cortex and lateral Cb in mutation carriers during sequence learning when compared with controls
Carbon 2010	USA	Case‐control	31	12	16−67	To measure sensorimotor activation at both the regional and network levels in carriers of the DYT1 dystonia mutation	11MDYT1 10NMDYT1	H2^[15]^O/**PET**	Increased NMRP in nonmotor task in all gene carriers. MDYT1 > NMDYT1 > CONTROLS In MDYT1, it correlated with severity of dystonia Increased NMRP during motor execution tasks in MDYT1 alone
Argyelan 2009	USA	Case‐control	28	8	15−44	To use magnetic resonance diffusion tensor imaging and probabilistic tractography to identify the specific circuit abnormalities that underlie clinical penetrance in carriers of genetic mutations for dystonia	7MDYT1 4NMDYT1 5MDYT6 4NMDYT6	DTI‐MRI	Reductions in cerebellothalamic connectivity correlated with increased motor activation responses, consistent with loss of inhibition at the cortical level. Nonmanifesting mutation carriers were distinguished by an additional area of fiber tract disruption situated distally along the thalamocortical segment of the pathway in tandem with the proximal cerebellar outflow abnormality. In individual gene carriers, clinical penetrance was determined by the difference in connectivity measured at these two sites
Carbon 2008a	USA	Case‐control	30	15	18−44	To explore whether PTD is associated with abnormal anatomical connectivity within motor control pathways	4MDYT1 8NMDYT1 3MDYT6	DTI‐MRI	Fractional anisotropy was significantly reduced in PTD patients in the pontine brainstem in the vicinity of the left superior cerebellar peduncle and bilaterally in the white matter of the sensorimotor region
Carbon 2008b	USA	Case‐control	12	6	9−43	To identify the neural substrates that support sequence learning in NMDYT1 mutation carriers	6NMDYT1	H2^[15]^O/**PET**	Increased cerebellar activation during sequence learning in DYT1 carriers
Draganski 2009	UK	Case‐control	79	28	19−76	To examine the relationship between dystonic phenotype and the DYT1 gene mutation by monitoring whole‐brain structure using voxel‐based morphometry	11MDYT1 11NMDYT1 15SPR+FHx 14SPR‐FHx	VBM	Non‐DYT1 adult‐onset dystonia patients and asymptomatic DYT1 carriers have significantly larger basal ganglia than healthy subjects and symptomatic DYT1 mutation carriers. There is a significant negative correlation between severity of dystonia and basal ganglia size in DYT1 mutation carriers
Vo 2013	USA	Case‐control	27	8	15−44	To make a group comparison of diffusion tensor imaging (DTI) results of dystonia patients and controls to reveal occult pathology	7MDYT1 3NMDYT1 5MDYT6 4NMDYT6	DTI	In dystonia mutation carriers, we detected fewer fibers in the cerebellothalamocortical pathways. This result agrees well with the findings of a previous study that used a probabilistic tractography method and demonstrated that gene carriers have less fiber tracts in the disease‐involved pathway
Premi 2016	Italy	Case‐control	43	26		To explore the complex perturbations in the different neural networks and the mutual interactions among patients with the DYT1 mutation	7MDYT1 10NMDYT1	VBM Rs‐fMRI	DYT1 mutation signature (symptomatic DYT1 and asymptomatic DYT1) was characterized by increased connectivity in the dorsal attention network and in the left frontoparietal network. Functional correlates of symptomatic DYT1 patients (symptomatic DYT1 vs. healthy controls) showed increased connectivity in the sensorimotor network
Sako 2015	USA	Case‐control	20	10	17−43	To explore whether the nonmotor manifestations of primary dystonia are associated with cerebellothalamocortical pathways circuit abnormalities	10MDYT1	DTI fMRI	The findings suggest that the normal pattern of brain activation in response to motion perception is disrupted in DYT1 dystonia. Thus, it is unlikely that the circuit changes that underlie this disorder are limited to primary sensorimotor pathways
Carbon 2011	USA	Case‐control	55	20	23−65	To determine whether there is increased cerebellar activation in clinically manifesting DYT1 carriers and in carriers of other primary dystonia mutations such as DYT6 To determine whether sequence learning performance and associated brain activation in these subjects correlate with previously described genotype‐related abnormalities of cerebellar pathway integrity and striatal D2 dopamine receptor binding	11MDYT1 13NMDYT1 7MDYT6 4NMDYT6	**DTI** [^11^C]‐raclopride/**PET** H2^[15]^O/**PET**	Affected DYT1 carriers exhibited significant increases in sequence learning‐related activation in the left lateral cerebellar cortex and in the right premotor and inferior parietal regions Increases in premotor cortical activation observed in the mutation carriers correlated with reductions in cerebellar pathway integrity measured using magnetic resonance diffusion tensor imaging and probabilistic tractography The cerebellar tract changes correlated with reductions in dentate nucleus activation recorded during task performance Sequence learning performance and task‐related activation responses did not correlate with striatal D2 receptor binding

MDYT1, manifesting DYT1; NMDYT1, nonmanifesting DYT1; MRI, magnetic resonance imaging; fMRI, functional magnetic resonance imaging; PET, positron emission tomography; DTI, diffusion tensor imaging; VBM, voxel‐based morphometry; FHx, family history; SPR, sporadic.

## 4 DISCUSSION

Most previous studies done on monogenic forms of dystonia, of which DYT1 is one, have explored pathophysiological mechanisms of dystonia using neurophysiological methods. Three main electrophysiological abnormalities implicated in the pathophysiology of dystonia have been elucidated. First is increased cortical excitability and deficient inhibition, including loss of surround inhibition at cortical, brainstem, and spinal cord level (Hallett, 2011) affecting all mutation carriers irrespective of clinical penetrance and affecting even the uninvolved body parts. This implies that additional factors, probably environmental, may be necessary to produce clinically eloquent disease (Edwards et al., 2006). The second is an exaggerated response to experimental interventions that induce plasticity in the sensorimotor system. This abnormal plasticity, which also affects clinically unaffected body parts, is not only lacking in NMDYT1, but they have a subnormal response to plasticity‐inducing experiments (Quartarone & Pisani, 2010). This suggests that the exaggerated response is linked to the clinically manifesting disease and that under‐activity may be protective. The third is the abnormal sensorimotor integration. As previously described, DYT1 mutation carriers show an abnormality in visual, tactile, and visuotactile discrimination, and the primary SMC, which processes temporal discrimination is the anatomical region responsible for this abnormality.

Neuroimaging studies have helped to delineate metabolic and microstructural abnormalities in dystonia patients and gene carriers, and most of the findings point to a disturbed connectivity in the CSPTC and CbTC pathways as highlighted in the findings from functional neuroimaging studies in the reviewed studies as highlighted in Table 1, and can be summarized as (1) increased metabolic activity in the lentiform nucleus, supplementary motor area, and cerebellum in addition (Carbon et al., 2004; Carbon et al., 2008a,b; Eidelberg et al., 1998; Ghilardi et al., 2003), (2) reduced striatal GABA and dopamine receptors (Carbon & Eidelberg, 2009; Garibotto et al., 2011), and (3) abnormal cerebellothalamocortical pathway (Carbon et al., 2004; Carbon et al., 2008a,b; Carbon et al., 2011; Draganski et al., 2009; Premi et al., 2016; Sako et al., 2015; Vo et al., 2013)

### 4.1 Metabolic abnormalities

#### 4.1.1 Abnormal glucose metabolism

In early studies of cerebral glucose metabolism, a genotype‐specific pattern of activation was described (Eidelberg et al., 1998). These changes, referred to as trait feature of the DYT1 carrier state, are expressed as a relative increase metabolism in the lentiform nucleus, cerebellum, and SMA present in all mutation carriers. This differs from what was described in the DYT6 carrier state, which is characterized by hypometabolism in the putamen and hypermetabolism in the temporal cortex (Carbon et al., 2004). Interestingly, manifesting carriers of both mutations showed a distinct pattern characterized by relative increases in the pre‐SMA and parietal association cortices and relative reductions in the cerebellum, brainstem, and ventral thalamus, the so‐called phenotype‐associated trait (Ghilardi et al., 2003). This pattern of activation may be necessary for the clinical manifestation of primary generalized dystonia, irrespective of the gene. Furthermore, these findings are consistent with the critical role of abnormal sensorimotor integration in the clinical penetrance of dystonia as indicated by metabolic abnormalities in the cortical regions involved in this process (Ghilardi et al., 2003).

#### 4.1.2 Suboptimal dopamine receptor binding

Dopamine has been found to play a role in some forms of secondary dystonia and dopa‐responsive dystonia. In vivo imaging studies have shown a moderate reduction in striatal D2 receptor binding in nongenetic forms of primary dystonia (Asanuma et al., 2005; Carbon et al., 2009; Naumann et al., 1998; Perlmutter et al., 1997), but its role in the pathobiology of DYT1 is not yet fully established even though Carbon and Eidelberg (2009) found a reduction in striatal D2 receptors among DYT1 carrier compared to healthy controls. In line with these findings in patients with sporadic disease, dopaminergic dysfunction evidenced by a reduction in the D2 receptor availability in the striatum in DYT1 mutation carriers compared to controls was reported by another group (Draganski et al., 2009). These reductions were significantly more pronounced in DYT6 than in DYT1 carriers, with no effect of clinical penetrance on radioligand binding. Overall, reduction in D2 receptor availability indicates that impaired dopaminergic neurotransmission can be mainly regarded as a trait characteristic of mutation carrier status although decreases in striatal D2 receptor availability have been found to correlate with increasing phenotype‐specific pattern of activation in a cohort of both DYT1 and DYT6 mutation carriers. Thus, although direct comparison of manifesting and nonmanifesting carriers did not reveal a robust effect of penetrance on striatal D2 receptor availability, the significant correlation between this measure and the expression of the penetrance‐related metabolic pattern suggests that abnormalities in dopaminergic transmission do contribute, to some degree, to the expression of clinical signs and symptoms. However, how it relates to microstructural abnormalities in DYT1 still needs to be worked out.

#### 4.1.3 GABA transmission

In vivo assessment of the GABAergic system in dystonia reveals a reduction in GABAa receptor expression and affinity both in DYT1 carriers (Garibotto et al., 2011) and sporadic patients in primary motor and premotor cortex, in primary and secondary somatosensory cortex, and in the motor component of the cingulate gyrus. This pattern of GABAergic receptor expressivity is not unique to DYT1 but also seen in other primary dystonias (Staege et al., 2021) and potentially leads to limited GABAergic synaptic transmission, neuronal disinhibition, and hyperexcitability.

#### 4.1.4 Harmonizing the roles of neurotransmitters

The role of the neurotransmitters dopamine and GABA is yet to be fully elucidated, but they do not appear to be innocent bystanders in the scope of events leading to the clinical manifestation of the disease. For example, GABA, apart from being the major inhibitory neurotransmitter, also has a role in modulating plasticity and, more interestingly, is affected by stress. This is in support of the *double‐hit theory* where there is a backdrop of a genetic susceptibility, representing the first hit, on which an external stressor works on (second hit), to produce clinically eloquent disease. It can thus be proposed that genetic predisposition leads to a neurodevelopmental abnormality of the CbTC pathway and hence neurochemical abnormalities in the basal ganglia and increased excitability of the cerebral cortex. A neurophysiological correlate of this model will be an abnormal sensorimotor integration due to the CbTC anomaly, leading to loss of inhibition and hence maladaptive plasticity.

From the neurotransmitter point of view, a reduction in the D2 receptors in the striatum would lead to an increased activation of the GABAergic inhibitory pathway to the GPe and then to a decreased activity of the inhibitory neurons from the GPe to the STN and consequently to an increased activity of the excitatory neurons from the STN to the GPi.

### 4.2 Microstructural abnormalities

#### 4.2.1 Abnormal sensorimotor activation

Further support for maladaptive sensorimotor processing has been provided by oxygen‐labeled H2O PET studies of DYT1 gene carriers tested in two scenarios (Carbon et al., 2010; Carbon et al., 2008a,b; Ghilardi et al., 2003). The first scenario assessed motor sequence learning and brain activation during a simple motor task (Carbon et al., 2008a,b; Ghilardi et al., 2003), and the other tested activation in a nonmotor audiovisual setting (Carbon et al., 2010). Not surprisingly, both manifesting and nonmanifesting carriers were comparable in their pattern of activation during nonmotor settings and exhibited genotype‐specific pattern of activation involving the brain regions mentioned above. However, there was a difference between the groups when they performed the motor tasks; only the manifesting carriers showed increased activation. This dissociation between motor and nonmotor pattern suggests an underlying processing abnormality in the sensory integration of audiovisual input and an additional motor‐activated dysfunction. However, it remains unknown whether increased motor cortical excitability and the disorganized sensorimotor integration reflect an intrinsic susceptibility to dystonia.

#### 4.2.2 Aberrant connectivity in the CbTC pathway

The demonstration of an aberrant connectivity in the CbTC pathway leads to abnormal sensorimotor integration and hence loss of inhibition and increased cortical excitability, which in turn causes a maladaptive neural plasticity. Since the abnormal connectivity in the CbTC appears to be the most compelling evidence of a neuroanatomical substrate that determines penetrance (Argyelan et al., 2009) and being a final common pathway, it may be proposed that it has a causal role to play in the chain of events leading to the clinical manifestation of the disease. The “protective polymorphism”−216H was also noted to be associated with a normal fractional anisotropy in this region, and when probabilistic tractography was applied, it showed a sparing of the proximal part of this pathway, which is affected in both MDYT1 and NMDYT1. In addition, changes in this pathway are also associated with activation changes in the SMC and SMA, which drives the sensory and motor abnormalities found in the dystonic brain.

How do these findings translate to the structural and functional basis of the disease? Abnormal plasticity is a function of the degree of inhibition, which perturbs sensorimotor integration. Abnormal sensory motor integration leads to a cascade of events, which under permissive factors, results in the clinical manifestation of the disease. Dystonia has been demonstrated to be associated with an abnormal somatotopic organization in the cortex coding the natural body part, which leads to a loss of the classic sensory homunculus and this finding has been correlated with clinical severity (Meunier et al., 2003).

There are two existing theories for the pathophysiology of DYT1 primary dystonia; first is the neurodegenerative theory based on the finding of ubiquitin and torsin A‐positive inclusion bodies in the brainstem (McNaught et al., 2004). Several factors make this difficult to sustain; this theory was based on only one study in which a small sample size and limited brain regions were studied. In addition, the age of onset of DYT1 primary dystonia does not fit into the typical age of onset of neurodegenerative disorders.

#### 4.2.3 Evidence for neurodevelopmental theory of dystonia

More studies have supported the neurodevelopmental theory (Carbon et al., 2010; Ghilardi et al., 2003; Naumann et al., 1998; Niethammer et al., 2011) (as opposed to neurodegenerative theory based on the findings of ubiquitin and torsin A‐positive inclusion bodies in the brainstem; McNaught et al., 2004). The age of onset of DYT1 primary dystonia in late childhood and early adolescence, when maturation of the thalamic and sensorimotor cortical pathways is known to occur, is the first pointer to a neurodevelopmental basis (Barnea‐Goraly et al., 2005; Gogtay et al., 2004; Paus, 2005).

Interestingly, neuroimaging studies have provided additional valuable evidence to support the neurodevelopmental theory. Developmental and morphological defects in the CbTC pathway have been demonstrated by DTI studies in DYT1 carriers, using probabilistic tractography. These findings have strengthened the basis for penetrance (Ghilardi et al., 2003; Naumann et al., 1998). The CbTC pathway appears to be the primary determinant of penetrance, as illustrated by the difference between manifesting and nonmanifesting gene carriers, as the latter has an additional fiber tract disruption in the distal part of the pathway, as discussed above. The abnormal transmission from the proximal portion does not make it to the basal ganglia and hence, the cerebral cortex. This abnormal connectivity may be a kind of protective loss of function in that regard. Interestingly, it was reported that the protective 216H polymorphism is associated with a normal CbTC connectivity, (Niethammer et al., 2011) suggesting that there is an established abnormality right from the outset, and who manifests the disease already has an imprinting.

#### 4.2.4 Multilevel loss of neuroinhibitory transmission

Loss of inhibition observed at multiple levels of the neuraxis on electrophysiology has been also supported by imaging techniques. This loss of inhibition is thought to be responsible for the cocontraction of agonist and antagonist muscles found in dystonia and is thought to be a function of an intact supraspinal control, particularly the corticostriatothalamocortical circuitry and the ability to select a wanted and unwanted movement. This has been also traditionally thought to be responsible for dystonic movement, but studies have shown that this abnormality is present even in nonmanifesting gene carriers. The CbTC has been noted to be the driver of increased cortical excitability and loss of inhibition in dystonia, as demonstrated by a concomitant increased activation of the SMC and SMA associated with disruption of this pathway. As earlier discussed, PET studies on receptor availability have demonstrated a reduction in GABA receptors in both focal dystonia and DYT1.

#### 4.2.5 Modulation of neuroplasticity

The cerebellum has been suggested to play an important role in modulating cortical plasticity (Luft et al., 2005; Meunier et al., 2003), such that abnormalities involving its outflow pathways may give rise to alterations in cortical responses during movement and learning and hence dystonia. The role of the cerebellum in learning has been demonstrated by several studies, and it is therefore not surprising that dystonics have an abnormality of sequence learning. This finding was confirmed by imaging studies where these patients could not recruit prefrontal cortices during sequence learning, a function controlled by the CbTC and associated connections. The role of VBM as a window to cortical plasticity is still in its infancy in DYT1, but a template for future research has been laid.

### 4.3 Hypothesis and future directions

Most of the studies using the different imaging techniques found abnormalities in the SMC, SMA, lentiform nucleus, and the cerebellum in both manifesting and nonmanifesting carriers of the DYT1 mutation. The abnormalities in these structures may represent a signature of the DYT1 mutation and could play a role in the pathophysiology of dystonia. Although it is difficult to implicate one of these microstructural abnormalities, as the primary driver of the pathophysiology from the available evidence, their findings can be harmonized into a unifying mechanism. It should be noted that most of these studies have not been replicated; of note are the ligand‐based studies. Nevertheless, the available findings can be a fulcrum for larger studies.

There are clear routes for future research: the first is to carry out more studies involving more heterogeneous and larger patient groups to establish the temporal‐causal relationship of the microstructural neurodevelopmental abnormalities versus the functional changes seen in DYT1 and the second is to use multiple imaging techniques in the same group of patients, for instance, PET, DTI, and VBM to link different findings both temporally and spatially. Many of the current studies used the “region of interest” approach, and so, specific regions of the brain were examined; this approach will not be adequate to study a disorder such as dystonia that affects a more widespread area of the brain. In this regard, VBM is a better choice of molecular imaging technique, because it provides a bigger evaluation window, but is not a replacement for others by no means.

## 5 CONCLUSION

With this review, we have attempted to propose a pathophysiology of primary generalized dystonia, using neuroimaging findings in DYT1 dystonia as a model. These findings support the view that dystonia is more of a network and neurodevelopmental disorder and demonstrates the potential role of imaging in adding to our understanding of the pathophysiology of this enigmatic disorder. Neuroimaging studies of a larger, preferably non‐heterogeneous group of patients, will extend the frontiers of our current knowledge.

## AUTHOR CONTRIBUTIONS

FTT performed the initial search and article screening while PBA double screened the articles. PBA and FTT wrote the initial draft. Both authors approved the final manuscript

## FUNDING

This work was not funded. None of the authors have received any funding from any institution, including personal relationships, interests, grants, employment, affiliations, patents, inventions, honoraria, consultancies, royalties, stock options/ownership, or expert testimony for the last 12 months.

## CONFLICT OF INTEREST STATEMENT

The authors declare no conflict of interest in this work.

## ETHICS STATEMENT

We confirm that no approval of an institutional review board or patients’ consent was required for this work. We confirm that we have read the Journal's position on issues involved in ethical publication and affirm that this work is consistent with those guidelines.

### PEER REVIEW

The peer review history for this article is available at https://publons.com/publon/10.1002/brb3.3023.

## Data Availability

The data that support the findings of this study are available from the corresponding author upon reasonable request.
